# The viability of utilising phone-based text messages in data capture and reporting morbidities due to lymphatic Filariasis by community health workers: a qualitative study in Kilwa district, Tanzania

**DOI:** 10.1186/s12913-022-08256-z

**Published:** 2022-07-19

**Authors:** Akili Kalinga, Michael Munga, Abdallah Ngenya, Winfrida John, William Kisoka, Ndekya Oriyo, Prince Mutalemwa, Wilfred Mandara, Leonard Masagati, John Ogondiek, Patricia Korir, Ute Klarmann-Schulz, Sacha Horn, Inge Kroidl, Alex Debrah, Achim Hoerauf, Upendo Mwingira

**Affiliations:** 1 National Institute for Medical Research (NIMR), Headquarters, Dar es Salaam, Tanzania; 2 Institute for Medical Microbiology, Immunology and Parasitology (IMMIP), Bonn, Germany; 3 Division of Infectious Diseases and Tropical Medicine, University Hospital Munich, Ludwig-Maximilians-Universität (LMU), Munich, Germany; 4 German Centre for Infection Research (DZIF), Neglected Tropical Diseases, Partner Site, Munich, Germany; 5 Kwame Nkrumah University of Science and Technology (KNUST), Kumasi, Ghana; 6 Research Triangle Institute (RTI), International, Washington DC, USA

**Keywords:** Mobile phone-based text messages, Mobile technology, Community health workers, Lymphatic Filariasis, Morbidity

## Abstract

**Background:**

Globally, there is recognition of the value of using mobile phones among health providers in improving health systems performance. However, in many Low- and Middle-income countries where there is shortage of health providers, Community Health Workers have assumed some responsibilities especially relating to identifying and reporting on health problems within their communities. Despite the known benefits of using mobile phone technology to deliver health services, there is limited information on the extent to which Community Health Workers are able to effectively use the technology in data collection and reporting. The aim of this study was to determine the feasibility of utilizing phone-based text messages on Lymphatic Filariasis morbidity surveillance by Community Health Workers.

**Methods:**

This was a cross sectional study whose data was collected through key informant interviews and focused group discussions among community health workers, health providers and staff of neglected tropical diseases control program. Collected transcripts were analysed through Thematic content analysis as it allowed for the identification of data codes through inductive reasoning.

**Results:**

The use of mobile phone-based text messages in data collection and reporting lymphatic filariasis morbidity cases by Community Health Workers was perceived by study participants to be a relevant tool and feasible due to the ease of use of the technology. The tool was reported by end-users to significantly increase their performance and efficiency was gained in terms of reduced paper work, increased the number of patients accessing health care services and the ability to report in real-time. All respondents were confident that Community Health Workers were the right persons to interact with communities in tracking and reporting morbidity cases using mobile technology.

**Conclusion:**

Mobile phone-based text messages have proven to be effective in routine workflows such as, data collection and reporting on Lymphatic Filariasis morbidity cases, patient to provider communication, decision making and supportive supervision. Mobile phones have also improved efficiency and general performance of end users in terms of increased number of cases identified and efficiency gained in terms of reduced paper work and the ability to collect and report in real-time.

## Background

Lymphatic filariasis (LF) is one of the most common disabling neglected tropical diseases (NTDs) targeted for elimination globally [1, 2] and in Tanzania [3]. Worldwide, around 51 million people are reported to be affected with LF [2]. LF is caused by roundworms namely *Wuchereria bancrofti, Brugia malayi and Brugia timori* which are transmitted between humans by mosquitoes such as *Anopheles, culex, Aedes and Mansoni* [4]. In 2000, LF infected about 120 million people in 72 countries, and about 40% of the burden of LF was in sub-Saharan African countries, including Tanzania. Approximately 1 billion people were at risk of contracting LF. In response, the World Health Organization (WHO) launched the Global Program to Eliminate LF (GPELF) in 2000 with the goal of eliminating LF as a public health problem by 2020 which is now revised to 2030 [2, 5, 6].

The parasites cause lymphatic vessel damage in infected people, which can lead to lymphedema and hydrocele. Without treatment, the disease can progress to elephantiasis, which causes poverty, discrimination, incapacity, discomfort, and social stigma [5]. Globally, 40 million people are estimated to suffer from LFrelated morbidities, with Tanzaniahaving 6 million people suffering from hydrocele and lymphoedema [6].

The WHO Road map (2021–2030) for neglected tropical diseases (NTDs) set a target of eliminating LF as a public health problem in order to achieve sustainable Developmental Goals (SDGs) through feasible, cost-effective approaches [7, 8]. For a country to be declared free of LF it has to enumerate people with LF morbidities and provide care to those patients [9]. Lymphoedema is managed through hygiene and skin care, wound care, exercise, elevation of the affected limb and proper footwear to prevent episodes of adenolymphangitis [10, 11]. Hydrocele can be managed through lymphatic draining or hydrocelectomy [12]. Globally, only 34 endemic countries actively implementing morbidity management and disability prevention (MMDP) services [12] and there are only seventeen countries including three countries from Africa which have been validated to eliminate LF as a public health problem [13].

Therefore, even if the transmission of the infection is largely stopped, there remains the issue of disease sequelae, such as lymphedema and hydrocele.

Tanzania adopted the Global Program to Eliminate Lymphatic Filariasis (GPELF) in 2000 under the Lymphatic Filariasis Elimination Program (LFEP). LFEP focuses on giving anti-filarial drugs to all eligible people in the endemic areas via mass drug administration (MDA) and provide management and prevent disability among those already having LF-morbidities [6, 14]. Although major achievements have been made in terms of interrupting LF transmission in 111 (93.3%) out of 119 filarial endemic districts in Tanzania, the management of LF major chronic manifestations (lymphoedema and hydrocele) has not been paid enough attention [15]. From 2017 to 2020, only a total of 6658 surgeries of hydrocele have been conducted countrywide while about 1134 people with lymphoedema have been trained and supported with hygiene kits to manage their conditions [15]. Unfortunately, there is no exact number of people suffering from both LE and hydrocele countrywide; it therefore, becomes difficult to calculate the proportion of individuals with LF morbidities.

The management of lymphoedema requires sustainable systems for tracking cases due to LF morbidities from remote areas where the disease is prevalent in order for them to access available care. In Tanzania, like many other endemic developing countries, there is a lack of a system to track cases of LF morbidities, especially in hard-to-reach areas [16].

The increased use of mobile phone technology has been shown to enhance the health systems in resource limited settings by improving data collection and reporting [17, 18]. Additionally, there is growing evidence pointing to the effectiveness of using mobile phone technology to increase the effectiveness of Community Health Workers (CHWs) in delivering services to the community members while at the same time improve the efficiency of data collection and quality of data [17, 19, 20]. Real time data collection and reporting of LF morbidity through mobile phone technology can be an essential resource in the LF management value chain [21]. The use of mobile phone technology to report LF morbidity cases using CHWs was piloted in Kilwa District, Lindi region and in Dar es Salaam and Mtwara regions using a text-based short messaging service (SMS) tool, [17, 22]. From pilot studies, the use mobile phone tool has helped to generate a close-to accurate estimate of LF morbidity data but has not yet been scaled up for nationwide use due to hindering factors. The assumed factors include over-reliance on paper-based collection and reporting by health providers, lack of trust of and reliability on mobile technology amongst the users, perceived technical difficulties among CHWs and health providers in using MP-BTMs and lack of understanding of the service delivery [23, 24].

### Community health workers: who are they and what are they doing?

The term “Community Health Workers (CHWs)” is used to describe a diversity of health aides who are selected and trained to work in their own communities. CHWs can be men or women, young or old, literate or illiterate [25]. Moreover, CHWs ensure community acceptance and ownership of existing health services [26]. Preventive chemotherapy for the roles and activities of CHWs are enormously diverse, within and across countries and across programs [27]. While in some cases CHWs perform a wide range of different tasks that can be preventive, curative and/or developmental, in other cases CHWs are appointed for very specific interventions [28, 29].

In Tanzania, in the context of supporting LFEP under the Neglected Tropical Diseases Control Program (NTDCP), CHWs have often been referred to as Community Drug Distributors (CDDs), a name that got fame initially from Community directed treatment with Ivermectin (CDTI) for control of Onchocerciasis and later with for five NTDs preventive chemotherapy using MDA [30]. In this country, CHWs perform a wide range of different tasks that can be preventive, curative and/or developmental (Home Based Care providers, Community-based distributors/educators, Para-social Workers for Orphans and Vulnerable Children, Peer educators, Peer counsellors, Community maternal, new-borns and child health care providers (MNCH), Life Skills Trainers, Traditional Birth Attendants). For the current two serial studies, CHWs were specifically used in their localities to identify and report LF morbidity cases using MP-BTMs. This emphasizes that the definition of CHWs must be contextualized to respond to local societal and cultural norms and customs to ensure community acceptance and ownership [31].

### The context

The healthcare sector in Tanzania relies largely on manual, paper-based patient records that are not centralised or integrated, creating communication barriers for different actors in the healthcare system. Within the LFEP, the routine activities of case identification, data collection, and reporting are done in collaboration with CHWs by health providers at primary level health facilities. The morbidity burden of LF in Tanzania has relied on the information from the MDA program where CHWs passively report cases identified annually at their localities. Consequently, the exact prevalence of morbidity cases has not always been accurate at the community or national level [22, 32].

Literature has shown the usefulness of mobile phones in improving health systems performance, increasing efficiencies and effectiveness in health care delivery in many low-income countries. For example, evidence shows that receiving text messages via mobile phones improves patients’ adherence to antiretroviral therapy and other clinical outcomes [17, 19].

Despite the known benefits of using mobile phone technology, there is limited information, in the context of NTDs, on whether CHWs are able to effectively use it to report the implementation of activities at the community level. Specifically, it is not clearly understood how CHWs perceives on the applicability and usefulness of mobile phone tools for data capture as compared to traditional ways of collection and reporting. Moreover, perceived lack of trust, reliability and technical difficulties to implement such mobile technology among CHWs and health providers in using MP-BTMs have not been elucidated before the system is scaled-up countrywide. Therefore, the aim of this study was to determine the feasibility of MP-BTMs by CHWs in data capture and reporting of cases of LF morbidities for them to access MMDP services provided by ministry of health through NTDCP and other partners.

## Materials and methods

### Study area

The study was conducted in Kilwa which is one of the six districts of Lindi region in Tanzania. Kilwa was purposively selected because is among the districts initially identified to have large number of LF cases and currently regarded as one of the hotspots for LF transmission (NTDCP, 2017)”. Since 2001, Kilwa has participated in MDA with Albendazole and Ivermectin for control of transmission of LF. The CHWs are involved in MDA activities at sub-community levels and reports to health providers who are staff of NTDCP at the nearest health facilities. In the recent sister study, CHWs were specifically trained by clinicians on how to quantify and report LF morbidity cases using mobile technology.

### Study design

This was a qualitative exploratory study whereby Key Informant Interviews (KIIs) and Focus Group Discussions (FGDs) were used to collect data. The selected design fits well within this study as it gives room for discussion of complex ideas in-depth, generates new ideas and promotes the exploration of the unknowns. The study was conducted between December, 2019 and January, 2020.

### Study participants

In this study, informants for KIIs and FGDs were purposively selected. Participants for FGDs were chosen among patients with LF morbidities and CHWs in their localities. Each ward in the district was represented by one randomly selected village where two (CHWs and patients) FGDs of either male or female participants were conducted depending on their availability. CHWs and patients were conveniently identified until the required number of 9–12 participants was exhausted in the sampled village.

Staff of NTDCP who were KIs were purposively selected from health facilities in the study districts and at national level. At each health facilities health providers responsible for MDA activities and trained on provision of MMDP services to LF patients, were purposively recruited for interview upon consenting. At national level, all programme officers who are responsible for training health providers on MMDP services were purposively selected for interview.

### Selection of study respondents

In this study, informants for KIIs and FGDs were purposively selected. Participants for FGDs were chosen among patients with LF morbidities and CHWs in their localities. Each ward in the district was represented by one randomly selected village where two (CHWs and patients) FGDs of either male or female participants were conducted depending on their availability. CHWs and patients were conveniently identified until the required number of 9–12 participants was exhausted in the sampled village.

Staff of NTDCP who were KIs were purposively selected from health facilities in the study districts and at national level. At each health facilities health providers responsible for MDA activities and trained on provision of MMDP services to LF patients, were purposively recruited for interview upon consenting. At national level, all programme officers who are responsible for training health providers on MMDP services were purposively selected for interview.

### Data collection methods

A total of 11 FGDs for both CHWs and LF patients of different sex were held after written informed consent was obtained from participants. Interview guide was used to collect responses from participants during discussions and covered important aspects such as CHWs perceptions, experiences and challenges faced in using MP-BTMs as data collection and reporting tool, and their perceived effectiveness in identifying LF cases and morbidity management, among others. Each group of either males or females was composed of 9 to 12 participants and discussions lasted for about 2 h. The discussions were facilitated by an experienced Social Research Scientists. Apart from notes taking, a Research Assistant was available to tape-record the discussions after obtaining consent from participants. Discussions were held at conveniently selected venue that ensured privacy and confidentiality.

A total of 16 Key informants were interviewed by experienced Social Research Scientists. Of these, 12 and four were staff of NTDCP at district and national levels respectively. Interview guide comprised issues also used for FGDs as described above. Each interview session took between 45 and 60 minutes at a conveniently selected venue that ensured privacy and confidentiality.

Guides for KIIs and FGDs were pre-tested prior to data collection and were conducted in Swahili which is the national official language spoken by more than 80% of Tanzanians. .

### Training of CHWs

CHWs who participated in this sub study were involved in the main study to identify and report cases with LF morbidities. Prior to being involved in the main study, CHWs were trained by study investigators on theoretical and practical aspects. They were trained on community sensitization, LF as disease, its mode of transmission, symptoms, preventive measures, severity (1-7stages) of Lymphedema and MMDP services. CHWs were also trained on data capture and the use of Unstructured Supplementary Service Data (USSD) technology for text messaging service.

### Data analysis

Qualitative data were analysed iteratively. All voice recordings from FGDs and interviews were transcribed and translated into English by research assistants. To check for accuracy, 10% of the transcripts were back translated into Swahili. Members of the research team then compared Kiswahili and English versions for differences and similarities while listening to the original voice recordings. Thematic Content Analysis (TCA) of transcripts followed transcription, allowing for the identification of multiple level of data coding [33] for analysis through inductive reasoning. Thematic Content Analysis (TCA) is a descriptive presentation of qualitative data as it portrays the ‘thematic Content’ of interview/FGD transcripts by identifying common themes in the text with contextual interpretation [34] The analysis commenced with the coding of transcripts, then organized and sorted by relevant themes for reporting. Sub-themes were also identified in the data through an iterative process, and codes were refined as needed during the analysis. Written notes from interviews complemented the transcripts. Data triangulation and verification occurred by comparing responses from different sources to identify similar themes and areas of (dis) agreement on issues across data sources and participants/respondents. An inductive approach to data analysis was used, guided by four questions based on the four pre-determined themes, namely: 1) perceived benefits of training prior to starting using mobile phones 2) CHWs/CDDs perspectives on their effectiveness in using mobile phones as data capturing and reporting tool 3) challenges faced by CHWs/CDDs in using mobile phones as data collection and reporting tool 4) Effects of training (if any) on using mobile phone technology as a data collection and reporting tool. Additional themes that emerged during analysis are also reported in the results section of this paper. Analysis of all the qualitative data from KIIs and FGDs was done by experienced social scientists namely MM, PM and WK.

### Trustworthiness

In this study we used various qualitative methods to qualify thematic content analysis. We intended to ensure that the findings obtained are credible, dependable, transferable and have authenticity. Therefore, throughout all stages from data collection to reporting of study results, we aimed to describe trustworthiness for the qualitative content analysis by both inductive and deductive approaches in the preparation organization, and reporting of study findings. Specifically, we performed triangulation and verification data by comparing responses from different sources. That is, authors used different techniques and sources of data collection. We used KIIs with NTD programme officers at district and National level and conducted FGDs with CHWs and individuals with LF morbidities for data/information collection and reporting. The use of two different methodological approaches and variety of study participants in collection of information for comparing and contrasting ensured dynamism in data sources and hence increased in credibility and authenticity of our findings.

## Results

### The effect of training of CHWs in enhancing data collection and reporting

It is important to note that before starting using mobile phones as a data collection and reporting tool, CHWs were engaged in training on how to use mobile phones as a tool to support data collection and reporting on LF morbidities. This section presents their experiences and perceived benefits of training prior to starting using mobile phones, perceptions on the feasibility, effectiveness and perceived challenges of using phone-based text messages in data collection and reporting LF morbidities.

It was almost unanimously conceded by KIs and majority of participants of the FGD sessions that the training helped the beneficiaries reduce errors in data collection and reporting hence enriched decision making at higher levels of the NTDCP. In fact, many further admitted that the training conducted helped CHWs to effectively and efficiently full fill their responsibilities and better on understanding procedures the patients should be doing to manage their conditions. CHWs themselves reported that training improved their approaches to communicate with communities and followed required steps especially when managing LE cases at community level. NTDCP officials reported on their perspective that through training, CHWs were able to track, identify, care and report individuals with morbidities due to LF in fastest way. As such, there was an increased number of patients reported at national level. Therefore, training has increased effectiveness in reporting and managing of LF Cases:

*“I can say the training was beneficial to us CHWs and to our patients because after training we were able to use the knowledge gained to sensitize LF patients to disclose and how to manage their conditions and therefore they have changed accordingly by coming out public”* (FGD female Participant, Mandawa)

Additionally, it was conceded by majority of FGD participants that stigma-reducing messages through sensitization and health education given to community by trained CHWs has made some patients accept their conditions and start seeking medical services something which was initially seen as unusual practice in the past.

### Effectiveness and feasibility of using MP-BTMs in data capture and reporting LF morbidities

Apart from discarding the need for them to carry manual registers during field visits, CHWs perceived a greater level of efficiency in routine reporting and record keeping especially when mobile phones were used albeit in tandem with manual registers. Generally, the use of mobile phones was perceived to be relevant and thus feasible for the day-to-day activities of CHWs. Further perception amongst CHWs is that the mobile phone-based text messages enabled a more direct channel of communication with senior staff of NTDCP at the district and national NTDCP level concerning status of LF morbidity cases.

*… “I think it is relevant and acceptable because it has simplified the reporting work, it is faster than paper-based reporting and the good thing these days almost everybody in the community has a mobile phone … ”* (Female FGD participant, Mandawa).

Another participant said that:

“*When reporting using papers, sometimes papers can get lost or torn especially when it is raining but with mobile phones it is easy and comfortable … .”* (FGD Female participant, Kilwa Masoko)

Participants from all FGDs conducted were in agreement that mobile phone technology has the potential to enhance community engagement through encouraging and simplifying the exchange of information between lower and higher levels of health program. Among the mostly cited advantages that were mentioned are real-time reporting of LF morbidity cases for timely decision making and enhanced CHWs-supervisor interactions. Respondents viewed the technology to be simple, fast and effective.

*… . “By using the Phone-based text messages for tracking LF morbidity cases, an instant message of the information required is received to confirm that the reported data has been received at the end”* (FGD Male participant, Mandawa).

Another participant had to say

*… “It is easy to use the menu in mobile phone because it is similar and familiar to one used for sending money and it only needs signals for mobile phone company to send text messages”* (Male FGD participant, Miteja).

It was learnt from both FGDs and KIIs that mobile phones are instrumental in facilitating referrals of the identified patients to access available MMDP services and remotely providing health education. The observation below further illustrates:

*.... CHWs had a perception that their effectiveness in case identification, referrals and provision of health education for morbidity management for LF patients has increased as a result of using mobile phone technology”.* (FGD Male participant, Tingi).

### Perceived impact of using MP-BTMs on performance of CHWs

The use of mobile phone-based text messages was said to enhance LF morbidity cases’ follow up by CHWs. That is, the ability to communicate with, tracking patients and reporting their information to national NTDCP level was enhanced and thus leading to improved performance. Generally, Many CHWs viewed the use of MP-BTMs in monitoring and reporting cases as very crucial in improving the performance of health care staff.

*“The phone helps in bringing patients information faster, and it helps to estimate new patients at the community.* (KII participant, Miteja).

Another participant responded that: -

*… “My experience with mobile phones, made my job easier and I can reach and connect clients to health services faster than before and I can monitor progress of their conditions even without coming into direct physical contact with them”.* (FGD female participant, Miteja).

Using mobile phone technology was further perceived as an effective tool for reaching many patients in a short time regardless of how remote and sparsely located they are.

*… “The mobile phone-based data collection and reporting system by CHWs helped in easy of registering new cases, reaching more LF patients and monitoring trends and reporting the disease without having national teams or LF coordinators at facilities to go looking for them.”* (KII participant, Kilwa).

The use of mobile phones among CHWs was perceived by majority of FGD participants to be associated with increased access to health care services and coverage of population. Health education provided by CHWs has brought up patients who would otherwise hide in their homes as a result of stigma or misconceptions about the disease and all its manifestations.

*… “using mobile phones is not ending at identifying and staging the patients but we also refer them to the health facilities for further action.”* (FGD male participant, Mandawa)

### Perceived contribution of CHWs in reduction of LF morbidity and transmission of disease

Positive perceptions by community members were demonstrated towards CHWs as having an important contribution in reduction of transmission of LF infections through tracking LF morbidity cases for them to access MMDP services and involvement in MDA activities at community level provided jointly by NTDCP and partners. CHWs were reported to have also involved in MDA activities at community level that contributes to reduction of transmission of LF. Therefore, CHWs contributes to curtailing disease transmission within their localities. One discussant put it:

*“In my opinion because they give us drugs through MDA and insist us to use bed nets consistently, that is the contribution of CHWs in transmission control”* (FGD male participant, Tingi)

Another discussant observed that since CHWs started using mobile phones, there has been noticeable improvement in the management of their conditions.

*“I think when they (CHWs) report our situation to higher authorities in time, those people respond immediately and we get attention accordingly”* (FGD female participant, Tingi)

### Perceived challenges experienced during case identification and reporting using MP-BTMs

The experienced challenges can be understood from three interrelated perspectives, namely; those of the LF patients, CHWs, and staff of NTDCP. Note that, the critical challenges are those perceived by CHWs who are the primary users of the digital technology in data collection and reporting of the LF morbidity cases as described here.

In this study, concerns were raised regarding challenges related to using mobile phone technology. The frequently cited challenge by the majority of KIIs and FGD participants was the mobile phone’s network instability especially in very remote areas.

… . *“One of the challenges which is not within our reach is network connection breakdown happening especially when you are submitting a report to the program officers at the national level; you may think it has gone but it takes time to reach the destination or sometimes it is not delivered altogether”* (FGD male Participant, Miteja)

It was further revealed that electricity was also critical for the smooth functioning of mobile phones. For mobile phones that need to be regularly charged, power black-out was frequently mentioned by the majority of FGD participants.

*… “Not every village has a stable electricity supply and network coverage. Hence if* power black-out *happens, CHWs may not be able to send the information to recipients until the systems stabilizes, this becomes a critical set back.”.* (FGD male Participant, Mandawa)

Another discussant said

*… . “Unreliability of electricity is quite challenging in our contexts. Sometimes one is forced to wait for 8 to 12 hours’ power black-out before power normalizes again. So, this makes it difficult to collect patients’ data and report them timely to the required authorities* (FGD Female participant, Kinyonga)

It was further learnt that the challenges that affect the LF clients and CHWs at the community level have a spill over effects on the operations of facility health staff at the primary health care level, district NTDCP program officials and finally affect the amount and quality of data captured at the national LF data repository. Accordingly, one informant revealed this:

*… “The NTDCP implements its activities using a cascaded bottom-up approach. Therefore, challenges on operations experienced at community and other lower levels in health care will definitely affect the data that is captured at the national level”*. (KII male participant, Kilwa).

## Discussion

The importance of including end users’ perspectives for effective implementation of mHealth interventions (e.g. MP-BTMs as analysed in this study) has been emphasized in the past [35]. However, the contribution of qualitative evaluations of the use of mobile phones to supplement the implementation of many mHealth interventions has not received much attention in many Low- and Middle-Income Countries (LMIC). This study adds important insights by presenting the CHWs’ (here in referred to as end-users of technology) perspectives on the effectiveness of MP-BTMs in data collection and reporting morbidities due to LF in a rural endemic district of Tanzania. This study also discusses the insights from CHWs in terms of its challenges and applicability in data capture and reporting of morbidities due to LF for future use in a rural endemic district of Tanzania.

Findings of this study have shown that CHWs have a positive perception that the uses of MP-BTMs increase their effectiveness and efficiency in routine reporting and record keeping of LF morbidities even when mobile phones are used in tandem with manual registers. CHWs have thus endorsed MP-BTMs in their day-to-day activities as useful and relevant. In line with the above findings, it is reported that CHWs in Uganda and Mozambique also felt that using mobile phone technology had the potential to improve their work efficiency, planning, and communication with supervisors [36].

Some of the encouraging results highlighted in this study include improvement of service delivery planning, time management and efficiency, accuracy of collected data, communication with supervisors, individual increased CHWs’ performance and accountability, and simplification of the work routine. This finding is in line with what has been reported in other studies [17, 20, 37]. Accuracy of the collected data is hereby emphasized as there were strong and positive perceptions by KIIs and FGD members that, compared to paper-based data collection, MP-BTMs for data collection has big potential in minimizing errors, it is user friendly and efficient in terms of saving time because information are shared in real-time [38].

The findings of this study corroborate the global literature and further point out that the use of mobile phone technology by CHWs has improved outreach services, data collection and management, and effectiveness in the reporting of morbidities [37, 39]. Moreover, mobile phone technologies are believed to have the potential to improve users’ knowledge, skills, and performance as reported elsewhere [17, 40–42]. Perceptions of CHWs in this study have also confirmed this revelation; they confessed to have improved in case finding, data management and reporting of LF morbidity cases when using MP-BTMs as compared to before using the technology.

Generally, the use of mobile technology in the delivery of health services, especially in remote settings where poverty related diseases are prevalent and where there is shortage of professional health providers is recommended for simplification of work, increasing accuracy and performance of staff who works for professional staff through task shifting. The use of mobile phone technology has proven effective and is associated with increased performance in various countries in Africa namely Ghana [43], Uganda [44] and Indonesia [45]. In Tanzania, it was previously studied that mobile phone short message service (SMS) reminders to CHWs promptness in making visits improved by 86% reduction in the number of days (9.7 to 1.4) for routine visits by CHW were overdue [46].

In this study, it was acknowledged by the majority of KIIs and members of the FGD sessions that the training provided helped the beneficiaries to reduce errors in data collection and reporting. Moreover, many LF patients and staff of NTDCP further admitted that the training helped CHWs to effectively and efficiently fulfil their responsibilities. As such, training played a main role fulfilling the role of tracking, managing and reporting in real-time, LF morbidity cases to national level and therefore enhanced decision making at the higher levels. The findings in this study is supportive of a study conducted in Rwanda that found that regular training of frontline health workers reduced the error rate for data entry from 54% at the start of the program to 8% over the course of 1 year [47]. Thus, it is important to provide sufficient initial and ongoing training to support the transition of workflow from a paper-based system to a digitized system [48].

Despite the positively perceived advantages of using MP-BTMs as demonstrated in this study, the innovation cannot go without implementation challenges. A number of studies suggest that CHWs encounter various challenges when using mobile health solutions such as mobile phones for health service delivery. These include lack of CHWs training on new mHealth solutions, weak technical support, issues of weak signal strength and other administrative related challenges. In this study, the mostly cited challenges were weak mobile signals which affected communication and unreliability of electricity was also repeatedly mentioned as a challenge as phones need to be sufficiently charged for all the mobile phone operations to go on smoothly. The findings presented in this paper especially those relate to challenges of phone charging due to unreliability of electricity from frequent power black-out and poor network coverage that were faced by CHWs agrees with the Ugandan and the Mozambican formative study [36].

## Conclusions

We conclude that MP-BTMs tool used by CHWs has proven to be effective in their routine workflows such as, data collection and reporting on LF morbidity cases, patient to provider communication, decision making and supportive supervision. The tool has also improved efficiency and general performance of CHWs in terms of increased number of cases identified and provided MMDP services, gains by reducing paper work and the ability to collect and report in real-time. Despite reported success stories of MP-BTMs, its implementation was hindered by weak mobile signals and unreliable electricity for charging mobile phones in remote settings that in turn affected smooth communication for data reporting. The applicability and importance of the findings of this study should extend beyond Kilwa district to other similar settings in the country and other LMIC.

## Data Availability

The datasets analyzed from this study for presentation of the results and concluding about the study are available on special request from r the corresponding author.

## Abbreviations

BMBF: Bundesministerium für Bildung und Forschung
CDD: Community Drug Distributor (CDD)
CDTI: Community Directed Treatment with Ivermectin
IMMIP: Institute for Medical Microbiology, Immunology and Parasitology
KNUST: Kwame Nkrumah University of Science and Technology
RTI: Research Triangle Institute
MDA: Mass Drug Administration
GPELF: Global Program to Eliminate Lymphatic Filariasis
LFEP: Lymphatic Filariasis Elimination Program
NTDs: Neglected Tropical Diseases
NTDCP: Neglected Tropical Diseases Control Program
WHO: World Health Organization
CHWs: Community Health Workers
NIMR: National Institute for Medical Research
